# Small-Pox

**Published:** 1872-03

**Authors:** 


### SMALL-POX

Has carried to the grave in Philadelphia, Pa., from the 1st day of September, 1871, to January 18, 1872, two thousand persons, — every single life a sacrifice to inexcusable negligence!

Before vaccination was found to be a protection against small-pox, thirty-five persons out of every hundred who had this disease died; now, only seven persons out of every hundred attacked, who have been vaccinated, so as to show good scars, die of small-pox or of its modified form.

If every infant is vaccinated in not less than four places at the same time, the liability to the disease is largely decreased; for if two of the four take, only four out of a hundred ever take the disease; if the four vaccinations take at once and well, less than one in a hundred take the disease. It is safer, more effective, and more certain to take the matter direct from the cow, though it affects the human system more violently; but after the virus of the cow has been applied to man, and that to another man, four or five times, it becomes milder.

Most persons have a sore arm from vaccination twice in a lifetime,—in infancy and at fifteen, if vaccinated well at that age; this ought always to be done; if it "takes" a second time, it will be good for life.

As a general rule, if vaccination takes well once, giving a large, well defined scar, the person is safe.

The older persons get after forty, the less liable do they become to small-pox, or varioloid, or any form of the disease. Vaccination has been practiced for seventy-five years, and in all the millions of cases in which it has taken, no educated physician has ever been able to discover that scrofula, syphilis, or any other form of disease has been conveyed to a vaccinated person; on the contrary, the mere fact of vaccination seems to render the human system less liable to attacks of ordinary diseases, just as persons who are strongly marked with small-pox are more healthy than they were before, and are less liable to other diseases; it seems to harden the constitution.

Many years ago it was suggested that if a person having the small-pox were placed in a dark room, there would be no pitting in the face. It is known that however severe the attack may be, and however deep and marked may be the pits on the face, no scar is ever seen on the scalp, under the hair; and the suggestion has been made that if the whole face was kept covered with common wax, during the presence of the disease, pitting might not take place at all. Some one has written to some of the papers that a perfect and safe

CURE FOR SMALL-POX AND SCARLET FEVER is found by dissolving one grain of sulphate of zinc, and one grain of digitalis or foxglove, and half a teaspoonful of brown sugar, in two tablespoons of water, and when thoroughly dissolved add four ounces more of water, that is, about eight tablespoonfuls; take one teaspoonful an hour. The reader is cautioned against paying any attention to such irresponsible statements; the best way is not to take the small-pox, by attending promptly to vaccination in your own person, and in those over whom you exercise authority. If you take small-pox, or if you have varioloid, that is small-pox made mild by previous vaccination, call in a physician of standing and skill, and commit yourself to him wholly. Besides, sulphate of zinc is a virulent poison; half a teaspoonful may kill a person; the diluted mixture above named, if taken at a single dose, would not be injurious; but a teaspoonful at a time, every hour, with no directions when to stop, might be taken so long as to poison the system as effectually as if a whole teaspoonful of the pure article were taken at a dose. If you would not attempt to tinker an old shoe that was not worth half a dollar, be persuaded not to tinker your constitution in the way of medicinal experiments; only the vulgar, the ignorant, or the penurious do such things.

